# Paracrine relationship between incretin hormones and endogenous 5‐hydroxytryptamine in the small and large intestine

**DOI:** 10.1111/nmo.14589

**Published:** 2023-04-03

**Authors:** Iain R. Tough, Mari L. Lund, Bhavik A. Patel, Thue W. Schwartz, Helen M. Cox

**Affiliations:** ^1^ Wolfson Centre for Age‐Related Diseases, Institute of Psychology, Psychiatry and Neuroscience King's College London Hodgkin Building, Guy's Campus London SE1 1UL UK; ^2^ The Novo Nordisk Foundation Centre for Basic Metabolic Research, Section for Metabolic Receptology and Enteroendocrinology University of Copenhagen Copenhagen DK‐2200 Denmark; ^3^ Centre for Stress and Age‐Related Diseases, School of Applied Sciences University of Brighton Brighton UK; ^4^ Present address: Chr. Hansen A/S, Human Health Research Hoersholm DK‐2970 Denmark

**Keywords:** 5‐hydroxytryptamine, enterochromaffin cells, gastric inhibitory polypeptide, glucagon‐like peptide‐1, motility, mucosal ion transport

## Abstract

**Background:**

Enterochromaffin (EC) cell‐derived 5‐hydroxytryptamine (5‐HT) is a mediator of toxin‐induced reflexes, initiating emesis via vagal and central 5‐HT_3_ receptors. The amine is also involved in gastrointestinal (GI) reflexes that are prosecretory and promotile, and recently 5‐HT's roles in chemosensation in the distal bowel have been described. We set out to establish the efficacy of 5‐HT signaling, local 5‐HT levels and pharmacology in discrete regions of the mouse small and large intestine. We also investigated the inter‐relationships between incretin hormones, glucagon‐like peptide‐1 (GLP‐1) and gastric inhibitory polypeptide (GIP) and endogenous 5‐HT in mucosal and motility assays.

**Methods:**

Adult mouse GI mucosae were mounted in Ussing chambers and area‐specific studies were performed to establish the 5‐HT_3_ and 5‐HT_4_ pharmacology, the sidedness of responses, and the inter‐relationships between incretins and endogenous 5‐HT. Natural fecal pellet transit in vitro and full‐length GI transit in vivo were also measured.

**Key Results:**

We observed the greatest level of tonic and exogenous 5‐HT‐induced ion transport and highest levels of 5‐HT in ascending colon mucosa. Here both 5‐HT_3_ and 5‐HT_4_ receptors were involved but elsewhere in the GI tract epithelial basolateral 5‐HT_4_ receptors mediate 5‐HT's prosecretory effect. Exendin‐4 and GIP induced 5‐HT release in the ascending colon, while L cell‐derived PYY also contributed to GIP mucosal effects in the descending colon. Both peptides slowed colonic transit.

**Conclusions & Inferences:**

We provide functional evidence for paracrine interplay between 5‐HT, GLP‐1 and GIP, particularly in the colonic mucosal region. Basolateral epithelial 5‐HT_4_ receptors mediated both 5‐HT and incretin mucosal responses in healthy colon.

## Graphical abstract

Enterochromaffin cell‐derived 5‐hydroxytryptamine (5‐HT) mediates gastrointestinal reflexes that are prosecretory and promotile. We determined the efficacy of 5‐HT signaling, local 5‐HT levels and 5‐HT pharmacology in discrete regions of the mouse small and large intestinal mucosae. The functional interrelationships between incretins, glucagon‐like peptide‐1 and gastric inhibitory polypeptide and endogenous 5‐HT upon mucosal ion transport, and motility were investigated.

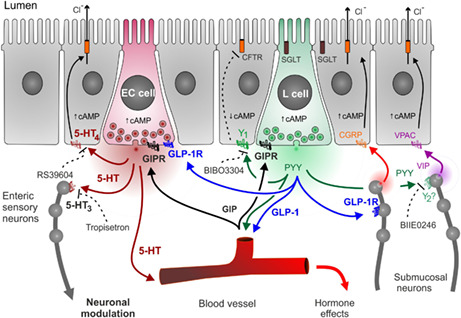

**Key points**

- Enterochromaffin cell‐derived 5‐hydroxytryptamine (5‐HT) mediates gastrointestinal reflexes that are prosecretory and promotile. Here we analysed the efficacy of 5‐HT signalling, local 5‐HT levels and 5‐HT pharmacology in discrete regions of the mouse small and large intestinal mucosae. The functional interrelationships between 5‐HT and two incretins, glucagon‐like peptide‐1 (GLP‐1) and gastric inhibitory polypeptide (GIP), on mucosal ion transport and motility were also investigated.
- Basolateral 5‐HT4 receptors mediated epithelial 5‐HT responses predominantly. Exendin‐4 and GIP stimulated 5‐HT release significantly in the proximal colon, which correlated with tonic 5‐HT activity and also with mucosal peptide responses that were partially mediated by 5‐HT. PYY also contributed to the anti‐secretory aspect of the colonic GIP response. Exendin‐4 and GIP slowed fecal pellet transit, the latter via a GLP‐1 mechanism.
- Functional interactions were identified between GLP‐1 and GIP, each of which enhanced local 5‐HT activities.

## 1 INTRODUCTION

Peripheral 5‐hydroxytryptamine (5‐HT, also known as serotonin) is produced and released in the gastrointestinal (GI) tract from two predominant sources, namely the enterochromaffin (EC) cells that produce ~90% of 5‐HT with the remainder of the amine thought to be released by intrinsic enteric neurons.^1^ EC cells are a prominent population of enteroendocrine cells observed along the length of the mammalian, including the mouse GI tract.^2^, ^3^ Despite the understanding gained over recent decades concerning the amine's origins, gaps still exist in our knowledge regarding the functional significance of endogenous 5‐HT activities within the GI tract.^4^ Of the multiple types of 5‐HT receptor, two are particularly important in the intestine; the G protein‐coupled 5‐HT_4_ receptor and the ligand‐gated cation channel, 5‐HT_3_ receptor, and both receptors are proven therapeutic targets. 5‐HT_4_ agonists such as prucalopride are pro‐kinetic and used to treat constipation of differing origins, including opioid‐induced constipation. 5‐HT also mediates vagal activation in emesis and 5‐HT_3_ antagonists (e.g. ondansetron) are proven anti‐emetic drugs. Aspects of 5‐HT mucosal signaling may also be luminally initiated^4^, ^5^ and thus nonabsorbable/GI restricted drugs targeted to receptors present on apical membranes may be efficacious.

Mucosal 5‐HT signaling is mediated predominantly by a combination of submucosal neuron 5‐HT_3_ and epithelial and/or neural 5‐HT_4_ receptors.^4^, ^6^, ^7^, ^8^ Activation of epithelial 5‐HT_4_ receptors causes electrolyte secretion^9^, ^10^ and neuronal 5‐HT_3_ activity can contribute to this response (in rat^11^) particularly when it is evoked by luminal cholera toxin.^10^ Endogenous 5‐HT released from EC cells also mediates mucosal responses to luminal nutrient‐derived and microbial metabolites such as short chain fatty acids.^12^, ^13^, ^14^, ^15^ Many of these same metabolites co‐activate other enteroendocrine cells such as L cells, that then release the incretin, glucagon‐like peptide‐1 (GLP‐1) and the anti‐obesity peptides, peptide YY (PYY) and PYY (3–36).^16^, ^17^, ^18^ Dietary nutrients such as long chain fatty acids or glucose stimulate L cell release of PYY and GLP‐1, the latter then causing 5‐HT release indirectly as a consequence of fat and glucose ingestion.^15^ Whether mucosal 5‐HT efficacy includes modulation of GI motility continues to be debated, with evidence for^19^, ^20^ and against^21^, ^22^ the initiation of colonic migrating motor complexes (MMCs).^5^ Nevertheless, 5‐HT_4_ agonists are prokinetic in man.^4^, ^23^

Enterochromaffin cells encompass a heterogeneous cellular population both anatomically (~10%) of EC cells exhibit luminal projections and basal extensions,^3^, ^24^ and phenotypically^13^, ^14^, ^25^ indicating that regional differences in 5‐HT signaling occur but are yet to be fully characterized. Given the accumulating evidence currently for EC cell involvement in GI chemosensation, one of our aims was to establish how endogenous 5‐HT signaling is modulated by hormones and luminal metabolites, in native mucosal preparations. Surprisingly this information is lacking. Notably, recent investigations have found that EC cells can be activated by incretin hormones, GLP‐1 and glucose‐dependent insulinotropic polypeptide (GIP),^15^ by noxious stimuli^26^ and by microbial metabolites.^27^, ^28^ Additionally, the amine can be secreted into the lumen where it may affect, or be altered by microbial metabolism^29^, ^30^, ^31^ as well as apparently activating host colonic luminal reflexes.^32^, ^33^ Furthermore, bacteria can produce tryptamine, a 5‐HT mimetic acting via 5‐HT_4_ receptors^34^ or via its indole catabolites that are proposed to stimulate TRPA1 present on EC cells to release 5‐HT in mouse and human intestine.^35^ Area‐specific differences in 5‐HT activities are evident^12^ although we^18^ and Martin et al.^12^ observed that luminal SCFAs do not appear to utilize EC cell‐derived 5‐HT directly in mouse colonic mucosa.

We propose that endogenous 5‐HT released from EC cells plays a significant role in the mucosal responses of the incretins GLP‐1 and GIP and, that colonic motility is also modulated by these peptides. The aims of the present study were to establish the area‐specific efficacy of endogenous 5‐HT (using competitive 5‐HT_3_ and 5‐HT_4_ antagonists) and to determine the 5‐HT levels in the same GI areas from the mouse. Identifying the epithelial sidedness of 5‐HT responses in the small and large intestinal mucosae was a further key objective. The expression and cellular localisation of GLP‐1 and GIP receptors in EC cells was also assessed in the duodenum and colon. The anti‐motility effects of GLP‐1 and GIP were also interrogated to determine whether 5‐HT_3_ or 5‐HT_4_ receptors were involved or modulatory in vitro or in vivo. These pharmacological investigations aim to clarify the inter‐relationships between EC cell‐derived 5‐HT, mucosal chemosensory mechanisms and motility in the mouse GI tract.

## 2 METHODS

### 2.1 Measurement of changes in mucosal preparation ion transport

Electrogenic ion transport was measured in mucosal preparations with intact submucosal innervation, from adult C57BL/6‐129/SvJ mice (12–20 weeks of age, either sex). Adjacent pieces of mucosae from different GI regions were prepared and voltage‐clamped at 0 mV in Ussing chambers, as described previously.^18^, ^36^ Vectorial ion transport was measured continuously as changes in short‐circuit current (*I*_sc_: μA/cm^2^) with intermittent recording of transepithelial resistance (TER). Investigations of 5‐HT pharmacology utilized the antagonists, ketanserin (a 5‐HT_2_ blocker, 1 μM, Sigma), tropisetron (5‐HT_3_ antagonist, 1 μM, Sigma) or RS39604 (5‐HT_4_ blocker, 1 μM, Tocris), followed by addition of 5‐HT (1 μM) as an internal control. Tegaserod (Tocris) was used as a 5‐HT_4_ agonist and a non‐cumulative concentration‐response curve was constructed for this agonist. An optimal blocking combination of 100 nM tropisetron plus 1 μM RS39604 was used to abolish endogenous 5‐HT signaling and revealed endogenous tonic 5‐HT activities. Neural blockade was achieved with tetrodotoxin (TTX, 100 nM, Sigma) as a pretreatment. Exendin‐4 (100 nM, Anaspec Inc.) was used to activate GLP‐1R^18^ while human (h)GIP (100 nM, Tocris) was used throughout to activate GIP‐R. Unless otherwise stated all peptide and drug additions were made to the basolateral reservoir. Endogenous GLP‐1R activity was blocked with exendin (9–39) (1 μM, Anaspec Inc.) while endogenous PYY (and NPY, both from Bachem) effects were abolished using a combination of the Y_1_ antagonist BIBO3304 (BIBO; 300 nM, Tocris) and Y_2_ antagonist BIIE0246 (BIIE; 1 μM, Tocris). Responses are the maximal changes from the equilibrated basal *I*_sc_ within 20 min of addition and these responses were pooled. To test whether 5‐HT uptake inhibition using the SSRI inhibitor, fluoxetine (10 μM, Tocris) would amplify endogenous 5‐HT signaling, we performed these experiments in tissues from PYY^−/−^ mice to avoid potential confounding inhibitory epithelial (or motility) effects of L cell modulation upon 5‐HT responses.^36^, ^37^

### 2.2 Measurement of 5‐HT in mucosal biopsies

Four adult C57BL/6J mice were killed by cervical dislocation and the entire GI tract excised. Mucosal biopsy punches (2 mm) were sampled from the duodenum, jejunum, ileum, ascending and descending colon. Each sample was homogenized and purified in ice‐cold 1 M perchloric acid, followed by precipitation of proteins by centrifugation at 14,000 *g* at 4°C. The supernatant was collected and run through an additional purifying step adding the same volume of 1 M perchloric acid, followed by re‐centrifugation. The protein‐free samples were diluted 10× in MilliQ water prior HPLC injection. Levels of 5‐HT were measured by HPLC‐ECD (HTEC‐500, Eicom) with a PP‐ODS2 column following the manufacturer's instructions regarding flow rate, mobile phase and applied potential. Retention time (3.7 s) and standard curves for quantification of 5‐HT, were determined using a control 5‐HT dilution curve.

### 2.3 Real‐time intestinal 5‐HT release measurements

Continuous amperometric measurements of 5‐HT overflow were performed on different GI segments (from C57BL/6J adult male mice, *n* = 4) that were pinned on a Sylgard® (Dow Corning) lined Teflon recording chamber and perfused with oxygenated Krebs buffer. The tissue was perfused for 10 min prior to baseline 5‐HT measurements, as detailed previously.^38^ In short, a boron‐doped diamond electrode with a potential of +650 mV oxidizes released 5‐HT that is then detected. This electrode was placed 1 cm from the tissue to measure background 5‐HT levels and using a micromanipulator, was then positioned 0.2 mm over the mucosa for 20 s in‐order‐to measure 5‐HT overflow. This was repeated three times before exendin‐4 (at 0.1, 1.0 or 10 nM, Tocris) or hGIP (100 nM, Bachem) were added and 10 min later, three additional 5‐HT measurements were obtained.

### 2.4 RNA in situ hybridization

Adult C57BL/6J male mice were euthanized by cervical dislocation and the whole GI tract was excised. Relevant intestinal segments were rinsed and fixed by luminal flushing with Bouin's fixative (50% ethanol and 5% acetic acid in dH_2_O). Thereafter, the intestine was opened and rolled around a toothpick to create a ‘Swiss roll’ preparation. These were fixed for a further 24 h in 4% paraformaldehyde and then paraffin‐embedded. Sections (5 μm thick) were cut on a microtome and dried onto glass slides. In situ hybridization was performed using RNAscope® 2.5 HD Duplex Assay (ACDbio) with probes for TPH1 (Probe‐Mm‐Tph1, #318701), GLP‐1R (Probe‐Mm‐Glp1r‐C2, #418851‐C2) and GIP‐R (Probe‐Mm‐Gipr‐C2, #319121‐C2) following the manufacturer's instructions. Visualization and image capture were performed using an upright Olympus BX51 light microscope with U‐RFL‐T light source and an Olympus DP71 camera.

### 2.5 Natural fecal pellet propulsion in vitro

Entire colons from adult C57BL/6J male or PYY^−/−^ mice were isolated and placed in Krebs–Henseleit buffer containing either 1 μM 5‐HT, 1 μM RS39604, 1 μM exendin‐4 or, 1 μM hGIP, and the movement of each pellet was noted by photographing colons at *t* = 0, *t* = 20, or *t* = 40 min when antagonist pretreatments were necessary. Data was pooled and analyzed as described previously.^36^

### 2.6 Total gastrointestinal transit time in vivo

On the day of experimentation C57BL/6J male mice (12–18 weeks old) were housed singly in cages with food, water, but a minimum of bedding. Four groups of 6 mice were administered by *i.p*. injection (10 mL/kg), with either vehicle (20% Tween80 in isotonic saline), exendin‐4 (3 μg/kg; Tocris), RS39604 and tropisetron (at 300 μg/kg and 1 mg/kg respectively; Tocris) or a combination of exendin‐4, RS39604 and tropisetron (at the same doses above). In some experiments hGIP (30 μg/kg; Bachem) was injected *i.p*. in place of exendin‐4. A 6% (w/v) carmine red suspension was prepared in 0.5% methylcellulose (both from Sigma‐Aldrich) in PBS. This was administered via oral gavage (250 μL) 15 min after drug administration. Individual mice were monitored for red pellet excretion at 20 min intervals. The time from gavage to the first appearance of a red fecal pellet was recorded as the total GI tract transit time.

### 2.7 Statistical analyses

Functional data observed at specified time points are expressed throughout as the mean ± SEM from a minimum of three specimens. Single comparisons between data groups were performed using Student's unpaired *t*‐test, whereas multiple comparisons used one‐way ANOVA with Dunnett's or Bonferroni's post‐tests as appropriate. *p* Values ≤0.05 were considered significantly different. For 5‐HT release and tissue levels, data sets were analyzed using a repeated measures 2‐way ANOVA with Tukey's post‐hoc test.

## 3 RESULTS

### 3.1 5‐HT_4_ activity predominates in mouse GI mucosae

**FIGURE 1:**
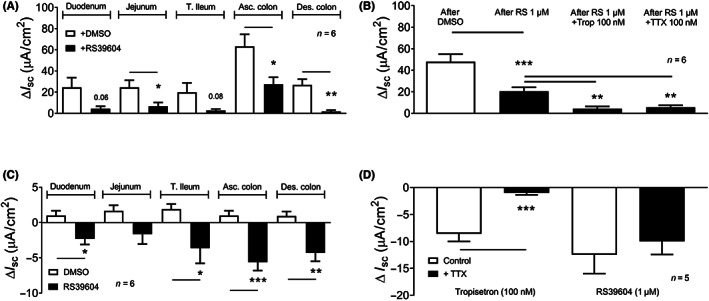
Mucosal 5‐HT responses and 5‐HT_4_ tonic activities in different areas of the mouse GI tract. 5‐HT (1 μM) responses after vehicle (DMSO, 0.01%) or 5‐HT_4_ antagonist (RS39604, 1 μM) addition to mucosa from, in (A) different GI regions and, in (B) the ascending colon where a combination of RS39604 and the 5‐HT_3_ antagonist, tropisetron (Trop, 100 nM) or tetrodotoxin (TTX, 100 nM) were added prior to 5‐HT. In (C) the effect of RS39604 (1 μM) or vehicle alone on basal *I*_sc_ levels reveals significant tonic 5‐HT_4_ activity in 4 of the 5 GI areas. (D) TTX pretreatment reveals sensitivity of tropisetron, but not RS39604 tonic inhibition of basal *I*_sc_ levels in ascending colon. Significant differences compared with respective controls are shown as: *p < 0.05, **p < 0.01, ***p < 0.001 using Student's *t*‐test (in A, C and D) or one‐way ANOVA with Dunnett's post‐test (in B). Bars are the mean ± 1 SEM from *n* = 5–6, as shown.

In mucosae prepared from the duodenum, jejunum, terminal ileum, ascending and descending colon, basolateral 5‐HT (1 μM) caused rapid elevations in *I*_sc_ (i.e. a secretory response) the peaks of which were pooled to provide the relative activities shown in Figure 1A. These responses were predominantly 5‐HT_4_‐mediated as pretreatment with the 5‐HT_4_ antagonist RS39604, significantly inhibited or abolished 5‐HT responses (Figure 1A) with the exception of the ascending colon where a 5‐HT_4_ resistant component was evident. Here, 5‐HT responses were abolished by inclusion of the 5‐HT_3_ antagonist, tropisetron (100 nM) with RS39604 (Figure 1B). Also, the combination of RS39604 and the neurotoxin TTX, abolished 5‐HT activity (Figure 1B). Notably, RS39604 alone lowered basal *I*_sc_ levels, revealing tonic 5‐HT_4_ activity that was significant in the duodenum, terminal ileum, ascending and descending regions of the colon (Figure 1C). Tropisetron also reduced *I*_sc_ in ascending colon mucosa and this 5‐HT_3_ tone was sensitive to TTX pretreatment whereas RS39604 was insensitive to neuronal blockade (Figure 1D).

**FIGURE 2:**
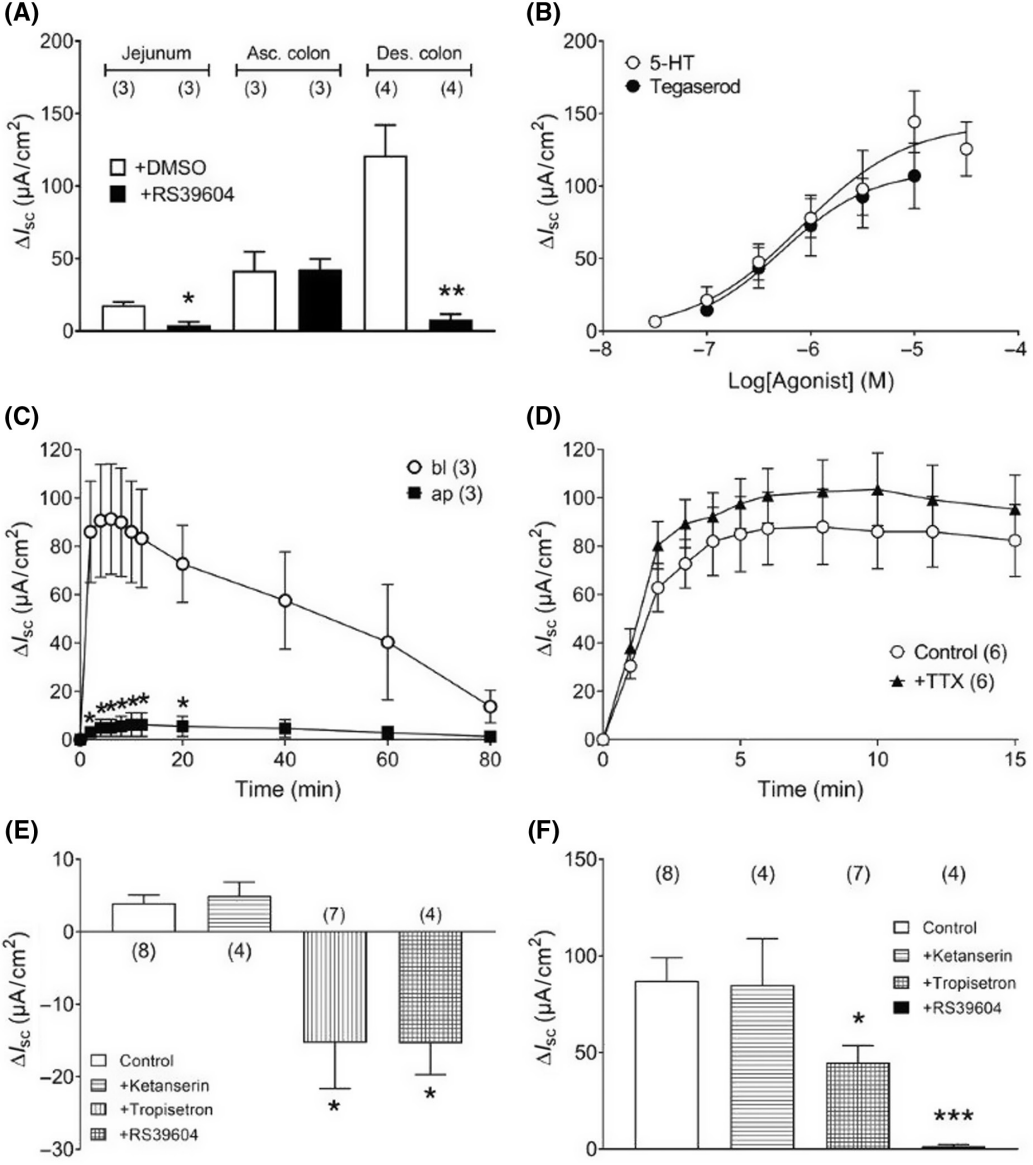
Tegaserod responses in three different GI regions (A) and 5‐HT pharmacology in mouse descending colon mucosa (B–F). In (A) tegaserod (1 μM) responses are inhibited by 5‐HT_4_ antagonist RS39406 (1 μM) in jejunum and descending colon, but not the ascending colon. In (B) concentration‐response relationships for basolateral 5‐HT or tegaserod in descending colon mucosa. In (C) time‐course comparison of basolateral (bl) versus apical (ap) 5‐HT (1 μM, added at *t* = 0 min) responses, in (D) basolateral 5‐HT responses are TTX (100 nM) insensitive. In (E) the effects of different 5‐HT receptor antagonists (each at 1 μM) on basal *I*_sc_ in descending colon mucosa are shown, and in (F) subsequent 5‐HT (1 μM) responses. Bars are the mean ± 1 SEM from *n* numbers shown in parenthesis. Significant differences compared with respective controls (*p < 0.05, **p < 0.01, ***p < 0.001) utilized Student's *t*‐test (A–D) or one‐way ANOVA with Dunnett's post‐test (in E and F).

The 5‐HT_4_ agonist, tegaserod (also known as HTF919; 1 μM) also caused rapid increases in *I*_sc_ and these secretory responses were sensitive to pretreatment with RS39604, except in the ascending colon where tegaserod responses were RS39604‐resistant (Figure 2A). In the descending colon mucosa where 5‐HT_4_ involvement predominated, tegaserod and 5‐HT exhibited similar potency (EC_50_ of 530 nM and 802 nM, respectively) and efficacy (Figure 2B). Notably, agonism was only evident after basolateral administration as shown by the *I*_sc_ time‐courses (Figure 2C), indicating that 5‐HT receptors are preferentially targeted to basolateral epithelial membranes and/or other cells in the lamina propria including submucosal neurons. The neurotoxin (TTX, which blocks neurotransmission within the intact submucosal innervation of these preparations^36^, ^39^) had no significant effect on 5‐HT (1 μM) responses in descending colon mucosa (Figure 2D) indicating that this is direct epithelial activity. Also, the 5‐HT_2_ antagonist, ketanserin did not alter basal colonic *I*_sc_ levels beyond vehicle control (Figure 2E) in contrast with the tonic activity revealed by the competitive 5‐HT_3_ or 5‐HT_4_ antagonists (tropisetron and RS39604) which each reduced *I*_sc_ levels per se. Subsequent 5‐HT responses were: (i) unaffected by ketanserin, (ii) partially inhibited by tropisetron, and (iii) were abolished by RS39604 (Figure 2F) confirming predominant epithelial 5‐HT_4_ signaling following exogenous 5‐HT administration to distal colon mucosa.

### 3.2 Co‐expression of TPH1 with GLP‐1R or GIPR in colon mucosa

**FIGURE 3:**
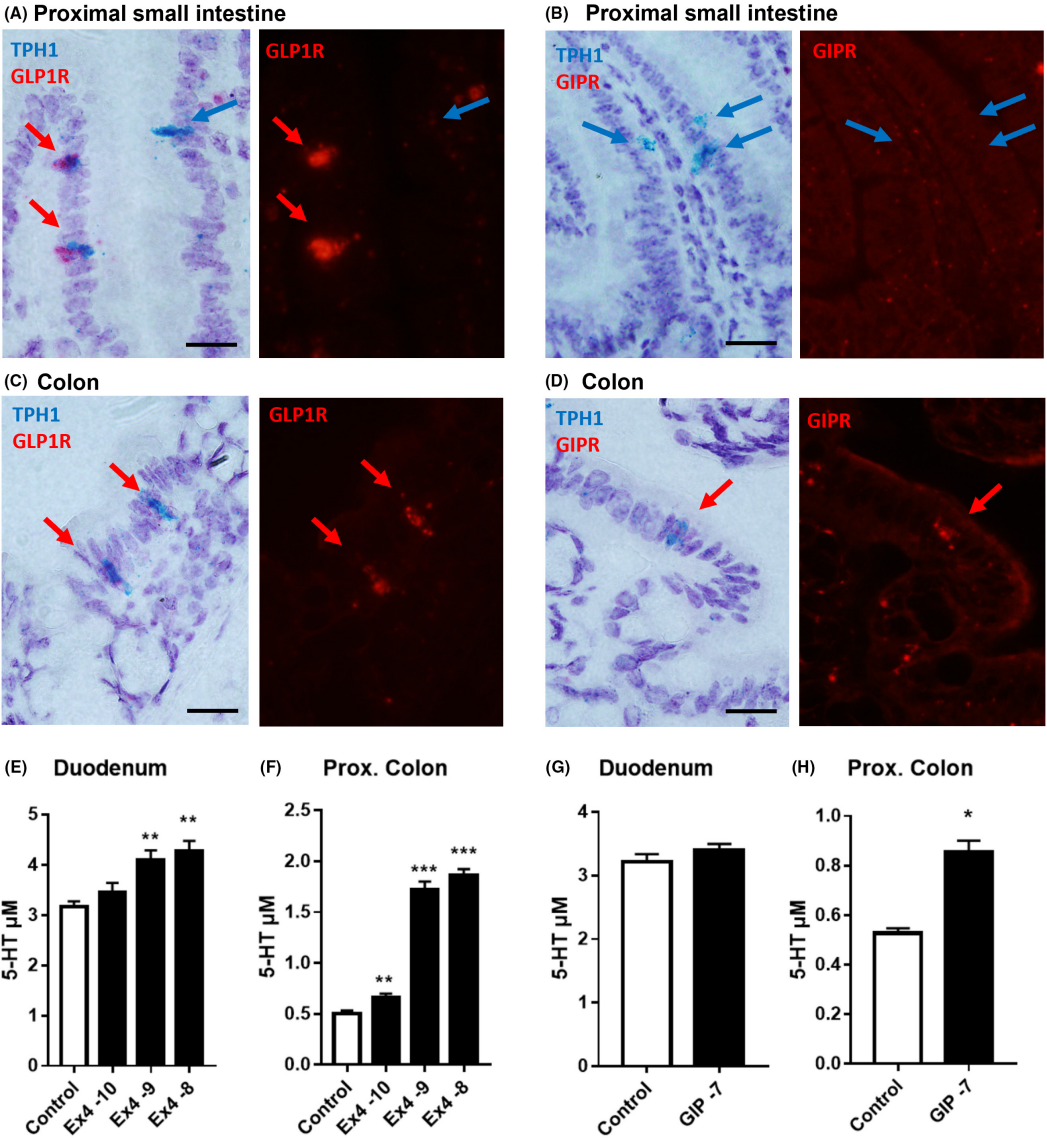
Expression of GLP1R and GIPR in EC cells (in A–D) and peptide‐induced 5‐HT release (in E–H). In situ hybridization for GLP1R (A and C) or GIP‐R (B and D) and TPH1 expression in proximal small intestine (A and B) and colon (C and D) mucosae. Blue arrows indicate TPH1 positive EC cells and red arrows indicate peptide receptor‐positive EC cells. Co‐localisation is observed in both GI regions for TPH1 and GLP1R (A and C), as well as between TPH1 and the GIPR in the colon (in D), but not between TPH1 and GIPR in the proximal small intestine (in B). Scale bars: 30 μm. Real‐time amperometric measurement of 5‐HT release from ex vivo duodenum (E and G) and ascending (proximal) colon (F and H) stimulated with GLP‐1 agonist: exendin‐4 (Ex4) (E, F) or hGIP (G, H) at the concentrations shown. Bars are the mean + 1SEM (*n* = 4 throughout) and statistical differences; *p < 0.05, **p < 0.01, ***p < 0.001 using repeated measures 2‐way ANOVA with Tukey's post‐test.

A previous study has shown GLP‐1R expression in EC cells located within the small and large intestine, while GIPR appeared to be co‐expressed with TPH1 but only in colonic EC cells.^15^ In the present study we observed a similar pattern of co‐expression between GLP‐1R and TPH1 in the proximal small intestine and in colonic EC cells (Figure 3A,C). However, in the duodenum (Figure 3B) GIPR and TPH1 were localized in different mucosal cells (Figure 3B shows three TPH1‐positive cells [left image] lacking labelling for GIPR [right image]). In contrast, some co‐localisation was observed for TPH1 and GIPR in colonic mucosa (Figure 3D, shows a single epithelial cell expressing both epitopes). These GI region‐specific differences in EC cell expression of peptide receptors were borne out by subsequent peptide‐induced 5‐HT release in the same GI areas. Notably, in mucosae from the duodenum and ascending colon we observed that the GLP‐1R agonist, exendin‐4 increased 5‐HT release significantly above basal levels (Figure 3E,F). In contrast, hGIP (at 100 nM) had no significant effect on duodenal 5‐HT release (Figure 3G), but it did cause significant amine release from ascending/proximal colon (Figure 3H). We also noted that basal levels of 5‐HT release were noticeably higher in duodenal, compared to ascending colon mucosae (Figure 3E–H).

### 3.3 Local levels of 5‐HT are highest in the ascending colon where tonic 5‐HT activity is greatest

**FIGURE 4:**
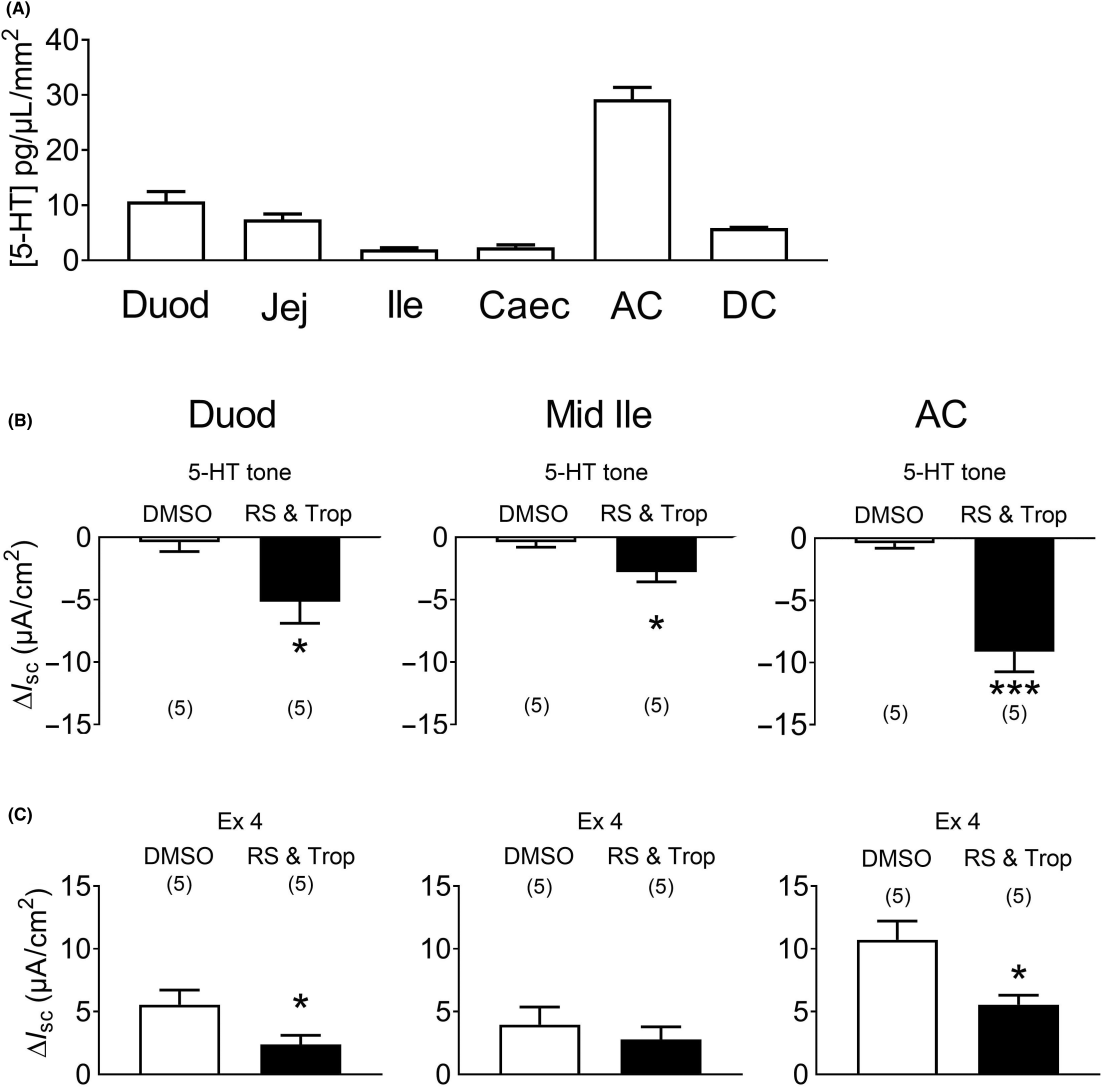
5‐HT content (A), tonic activities (B) and subsequent exendin‐4 responses in different regions of the mouse GI tract (C). In (A) mucosal 5‐HT levels were established using HPLC‐ECD. Values are the mean + 1SEM (*n* = 4). Duod, duodenum; Jej, jejunum; Ile, ileum; Caec, caecum; AC, ascending colon; and DC, descending colon. In (B), tonic 5‐HT_3_ and 5‐HT_4_ activity revealed using a combination of 5‐HT_3_ and 5‐HT_4_ antagonists (1 μM RS39406 and 100 nM tropisetron: RS & Trop) added basolaterally. In (C), subsequent exendin‐4 (Ex 4, 100 nM) responses were partially inhibited by RS and Trop. Bars are the mean ± 1 SEM, from numbers in parenthesis. Significant differences compared to vehicle controls (0.1% DMSO) are shown (*p < 0.05, ***p < 0.001) using Student's *t*‐test.

Punch biopsies of mouse mucosae from different GI regions revealed the highest 5‐HT levels were present in the ascending colon followed by the duodenum, the jejunum, descending colon, caecum and finally the ileum, which exhibited the lowest levels (Figure 4A). This correlated with the relative levels of 5‐HT_3_ and 5‐HT_4_ tonic activities revealed by addition of a combination of 5‐HT_3_ and 5‐HT_4_ antagonists, tropisetron and RS39406 respectively (Figure 4B). Since exendin‐4 stimulated significant 5‐HT release from duodenal and ascending colon preparations (Figure 3E,F), we set out to establish whether 5‐HT mediated a proportion of exendin‐4 mediated increases in *I*_sc_ in this proximal colonic region.^40^ Exendin‐4 raised *I*_sc_ levels as previously described^40^ and these rapid responses were partially inhibited by pretreatment with the 5‐HT_3_ and 5‐HT_4_ blockers, significantly so in the duodenum and ascending colon (Figure 4C) indicating that activation of GLP‐1R on EC cells results in 5‐HT release and increases epithelial *I*_sc_ as a consequence. Subsequent 5‐HT responses were abolished by the 5‐HT antagonists in each GI area (*data not shown*, but as seen previously in Figure 1A,B).

### 3.4 GIP mucosal responses involve a combination of endogenous 5‐HT and PYY

**FIGURE 5:**
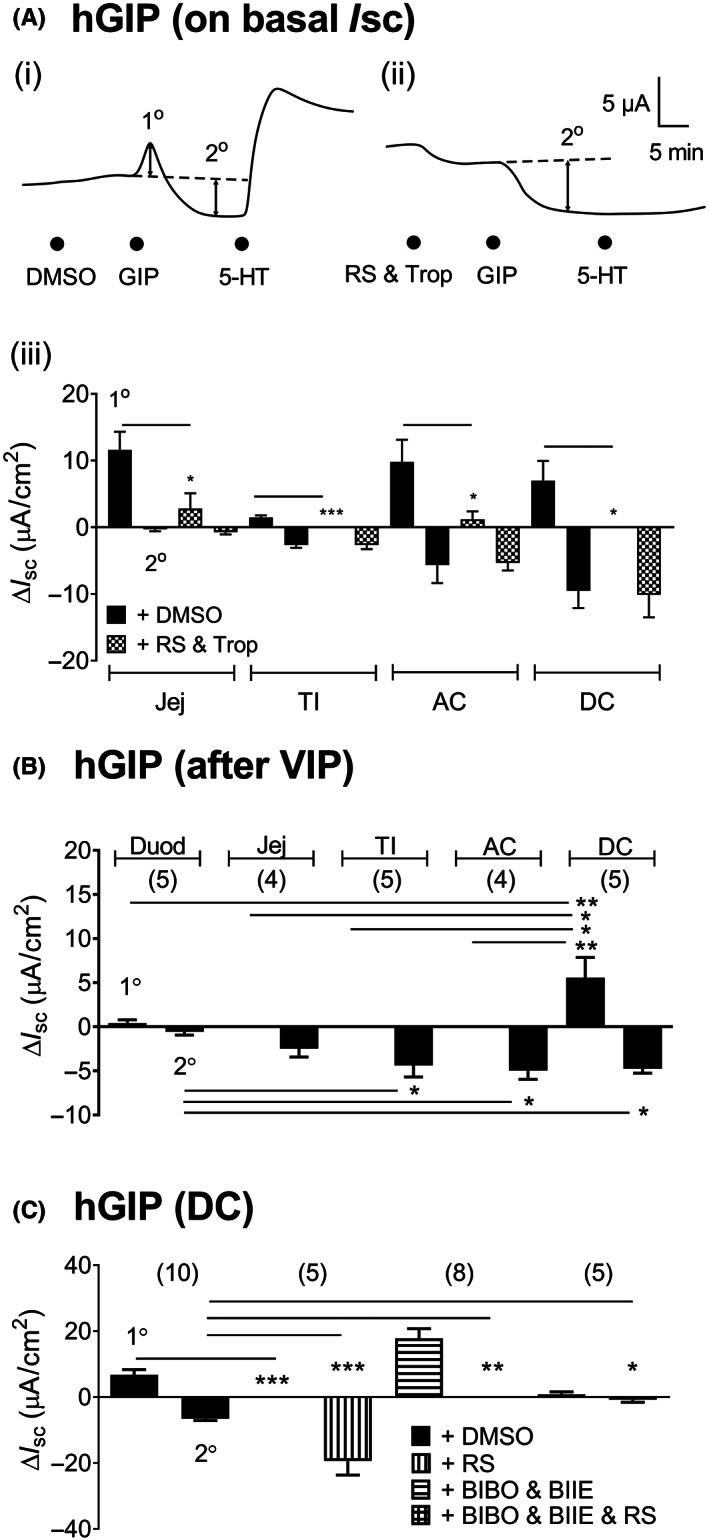
Mucosal hGIP signaling under basal (A) and VIP‐stimulated (B and C) conditions. In (A) example traces showing biphasic hGIP (100 nM) responses in (i) vehicle‐treated colon mucosa and, (ii) following 5‐HT antagonists (RS39604, 1 μM) and tropisetron (100 nM, RS & Trop) in ascending colon mucosa. In (iii) pooled data showing the 1° and 2° components of the hGIP *I*_sc_ response in different GI regions (jejunum [Jej], terminal ileum [TI], ascending colon [AC] and descending colon [DC]) and their sensitivity to RS & Trop (*n* = 6). In (B), hGIP responses (100 nM) after pretreatment with the secretagogue, VIP (10 nM) were biphasic (1° and 2° components) in different GI regions, particularly so in descending colon (DC). In (C), the descending colon hGIP 1° response is abolished by 1 μM RS39406 (+RS) while the hGIP 2° response is PYY‐Y_1_ and Y_2_‐mediated (abolished by 300 nM BIBO3304 and 1 μM BIIE0246; + BIBO & BIIE). All three blockers RS39406, BIBO3304 and BIIE0246 (+ BIBO & BIIE & RS) abolished the biphasic hGIP response. Bars are the mean ± 1 SEM, and significant differences compared with vehicle (0.1% DMSO) controls are shown (*p < 0.05, ***p < 0.001; used Student's *t*‐test here). In B and C (where *n* numbers are shown in parenthesis) significant differences compared to respective controls are shown (*p < 0.05, **p < 0.01, ***p < 0.001) using one‐way ANOVA with Dunnett's post‐test.

In contrast with the monophasic exendin‐4 responses, mucosal hGIP responses were biphasic in preparations of small and large intestine. In ascending colon mucosa (Figure 5A (i)), hGIP caused an initial increase in *I*_sc_ (1° component) that was abolished by 5‐HT blockers (Figure 5A (ii)) leaving a slower reduction in *I*_sc_ to hGIP (2° component; Figure 5A (i) and (ii)). Similar biphasic effects were observed in colonic mucosa with mGIP (100 nM, *data not shown*). A survey of four GI regions revealed that hGIP acute signaling in untreated mucosae was greatest in the jejunum, ascending and descending colon (Figure 5A (iii)) and was less significant in the terminal ileum. Unexpectedly, 5‐HT antagonists significantly reduced the transient 1° hGIP response in all four GI regions (Figure 5A (iii)) leaving the 2° reductions in *I*_sc_ unchanged. Since GIPR are expressed by L cells^15^ and the frequency of these enteroendocrine cells increases distally in the colon,^2^ we next investigated whether hGIP co‐activated L cells as well as EC cells in the descending colon. Here, we also pretreated colonic mucosa with the secretagogue VIP, to optimize L cell PYY G_i_‐coupled epithelial signaling.^36^, ^37^ As expected, VIP raised *I*_sc_ levels in all GI regions (*data not shown*) and 10–15 min later when this epithelial secretory response had stabilized, the 1° component of hGIP response was absent from all but the descending colon mucosa (Figure 5B). In this distal region of the colon, pretreatment with RS39604 also abolished the 1° hGIP response and consequently increased the slower 2° reduction in *I*_sc_ (Figure 5C). The latter was selectively inhibited by a combination of PYY‐Y_1_ and Y_2_ antagonists (BIBO and BIIE) indicating involvement L cell‐derived PYY in the 2° phase of the GIP response. A combination of 5‐HT_4_, Y_1_ and Y_2_ antagonists abolished both 1° and 2° aspects of the hGIP *I*_sc_ response (Figure 5C). Subsequent 5‐HT responses were abolished by RS39406, while PYY antisecretory responses (10 nM^36^) were abolished by the Y_1_ and Y_2_ antagonists (BIBO3304 and BIIE0246; *data not shown*).

### 3.5 Comparison of pro‐motile 5‐HT activity and anti‐motile GLP‐1 and GIP effects in mouse colon in vitro

**FIGURE 6:**
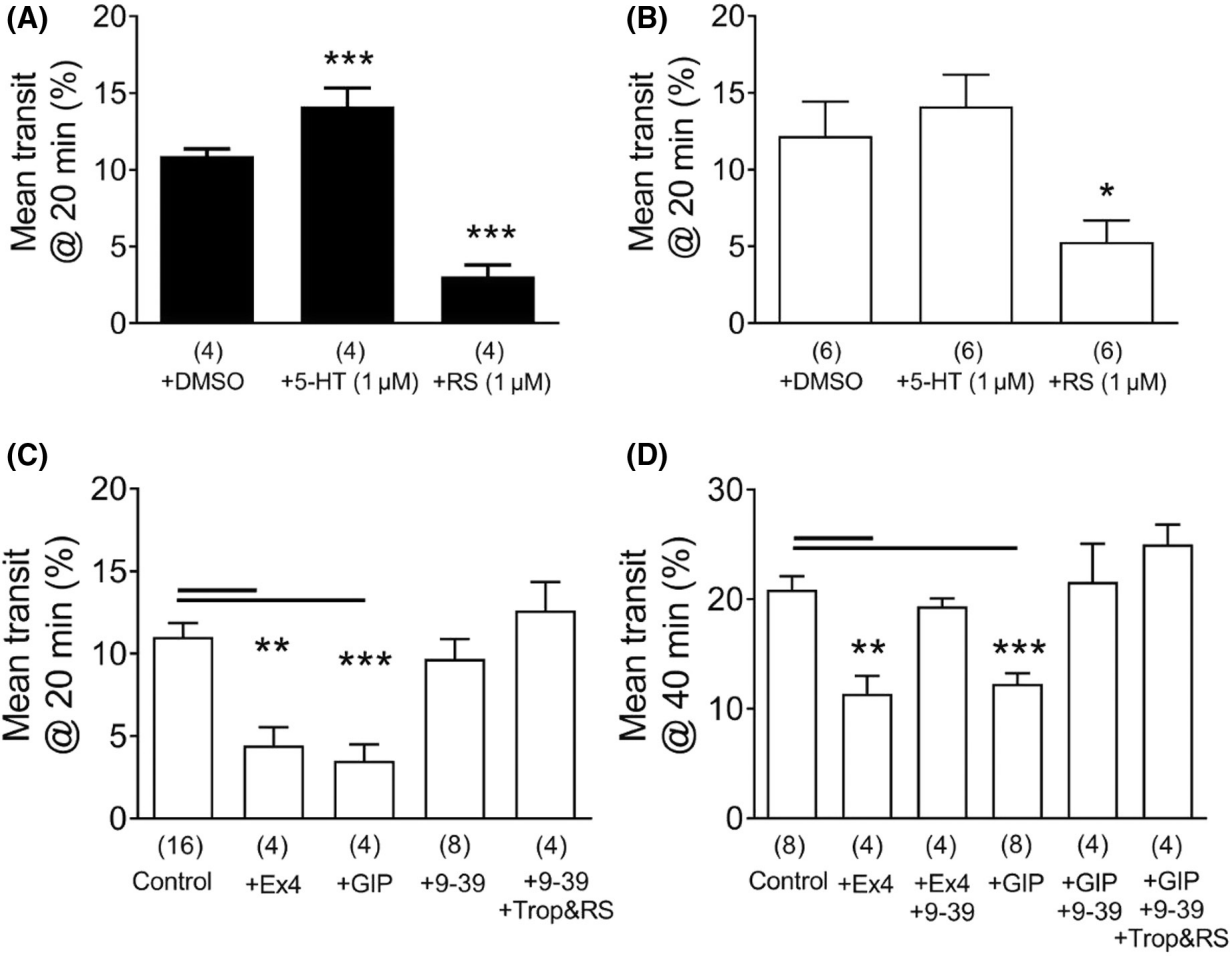
Natural fecal pellet transit in WT (in A) and PYY^−/−^ (in B–D) colons in vitro. In A and B similar sensitivities to 5‐HT or the 5‐HT_4_ antagonist RS39604 (at concentrations shown) increase or decrease motility, respectively. In (C), the effects of vehicle (controls) or exendin‐4 (+Ex4, 100 nM), hGIP (+GIP, 100 nM), the GLP‐1 blocker exendin (9–39) (+9–39, 1 μM) alone or with the 5‐HT_3_ and 5‐HT_4_ antagonists (1 μM RS39604 and 100 nM tropisetron: +Trop&RS) on transit over a 20 min period. In D (note the different y axis), the anti‐motile effects over a 40 min period to Ex4 or hGIP (alone, at the same concentrations used in C) or after Ex (9–39) alone (+9–39) or with tropisetron and RS39604. Each bar is the mean + 1 SEM from *n* numbers shown in parenthesis. Statistical differences from vehicle controls are as shown (*p < 0.05, **p < 0.01; ***p < 0.001) using one‐way ANOVA with Dunnett's post‐test.

Given the involvement of endogenous 5‐HT in the secretory components of colonic mucosal GLP‐1R and GIPR responses, our next aim was to determine whether an inter‐relationship existed between GLP‐1 and GIP's anti‐motility effects and endogenous 5‐HT signaling in WT, or in PYY^−/−^ mouse intestine. PYY is co‐localized with GLP‐1 in L cells,^2^ and previous studies utilizing PYY^−/−^ mice have allowed the functional effects of GLP‐1 agonism to be more readily interrogated in the absence of PYY that is known to mediate both ileal and colonic brakes. Natural fecal pellet propulsion in vitro was increased by 5‐HT, significantly so in WT colon (Figure 6A). Transit was also accelerated slightly by 5‐HT in PYY^−/−^ colon, but this was not statistically significant (Figure 6B). As expected, the 5‐HT_4_ antagonist RS39604 slowed fecal movement significantly in WT and PYY^−/−^ colon (Figure 6A,B). Both exendin‐4 and hGIP slowed pellet transit significantly (Figure 6C). GLP‐1 antagonism (exendin (9–39)) alone, or in combination with tropisetron and RS39604, had no significant effect on fecal pellet movement over a 20 min period (Figure 6C). GLP‐1R antagonism did however, reverse the anti‐motile colonic effect of exendin‐4 and notably also of hGIP (Figure 6D). The blockade of endogenous 5‐HT mechanisms by inclusion of tropisetron and RS39604 with exendin (9–39) did no more than the GLP‐1R blocker alone. Exendin (9–39)'s ability to reverse exendin‐4's and hGIP's anti‐motile effects, indicates that GLP‐1 mediates GIP‐retarded colonic transit. Endogenous 5‐HT appears not to be involved in either peptide effects in isolated mouse colon.

### 3.6 Exendin‐4 retards whole GI transit in vivo while GIP is inactive

**FIGURE 7:**
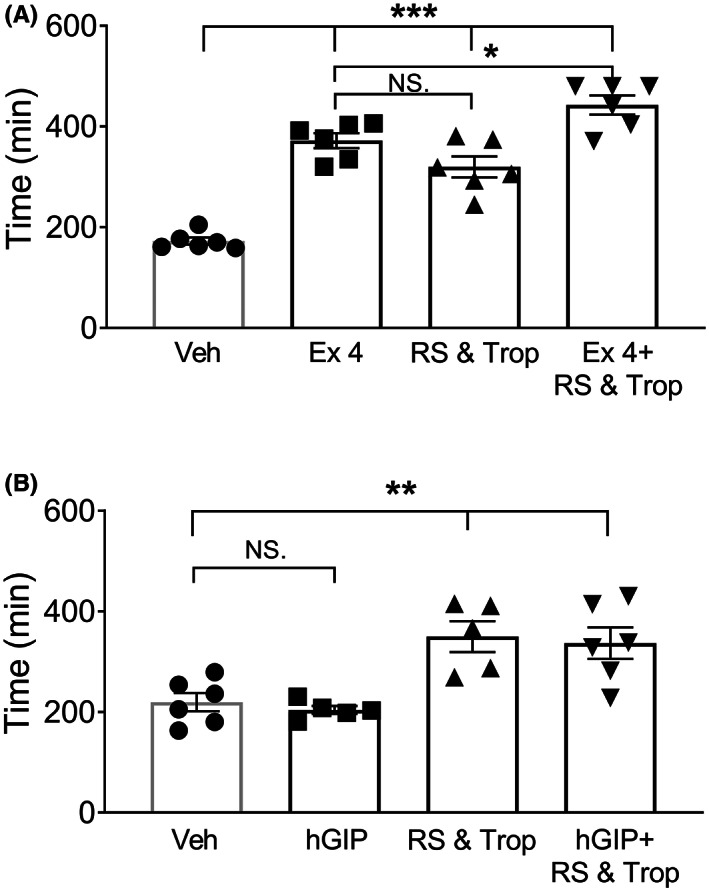
Total GI tract transit time is enhanced by GLP‐1R activation or by 5‐HT_3_ and 5‐HT_4_ blockers, but is unchanged by hGIP. Oral administration of carmine red enabled full‐length GI transit time to be measured after *i.p*. injection of GLP‐1R (Ex‐4, in A) or GIPR agonist (hGIP, in B). In (A), exendin‐4 (Ex‐4: 3 μg/kg), or hGIP (in B, at 30 μg/kg), or the combination of 5‐HT_3_ antagonist, tropisetron (Trop: 1 mg/kg *i.p*.) and 5‐HT_4_ antagonist, RS39604 (300 μg/kg *i.p*.) were administered ± either peptide. Bars are the means ±1 SEM from *n* = 6 mice throughout. NS. denotes non‐significance, while statistical differences are shown as; *p < 0.05, **p < 0.01, ***p < 0.001 (using 1 way ANOVA with Tukey's post‐test).

**FIGURE 8:**
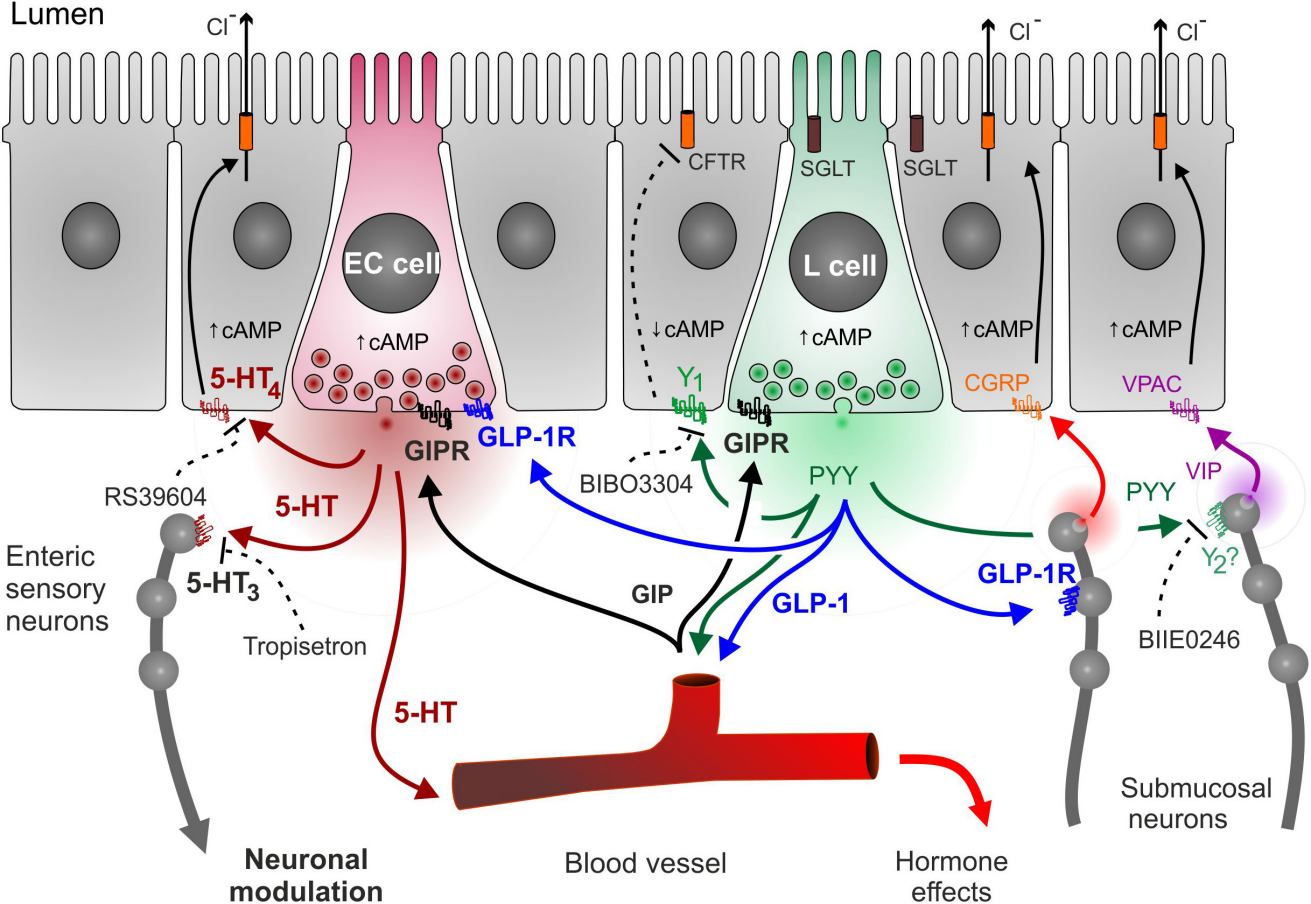
A schematic showing local 5‐HT, GLP‐1 and circulating GIP signaling in mouse colonic mucosa. Responses mediated via enterochromaffin (EC) cell 5‐HT, and L cell derived peptides, PYY and GLP‐1 are summarized. PYY (in green) and GLP‐1 (blue) mechanisms with GIP (black) and their respective receptors (blocked by selective antagonists, as shown). The *I*_sc_ changes we observed result predominantly from changes in epithelial electrogenic Cl^−^ secretion, mediated primarily via cAMP‐sensitive CFTR located on apical membranes (orange barrels). Modulation of submucosal neuron activities are included as indicated by our functional studies.

Having established that exendin‐4 and hGIP slow fecal pellet progression down the isolated colon we next examined whether their anti‐motile effects were present along the length of the GI tract in vivo. Whole GI transit time was doubled by exendin‐4 alone and prolonged significantly by the combination of tropisetron and RS39604 (Figure 7A). The 5‐HT antagonists additionally prolonged transit in the presence of exendin‐4 (Figure 7A). In contrast, hGIP at 30 μg/kg (an *i.p*. dose known to have efficacy^15^) had no effect on GI transit time (Figure 7B). Within this data set the combination of 5‐HT_3_ and 5‐HT_4_ blockers retarded motility to the same degree with or without hGIP (Figure 7B), indicating that hGIP was not modulating tonic 5‐HT pro‐motile effects significantly along the length of the GI tract. Thus, in healthy mice the GLP‐1/GIP and 5‐HT motility pathways appear to be independent while, in contrast, significant dependence of GLP‐1 and GIP responses upon endogenous 5‐HT exists at the mucosal level (as shown in the schematic, Figure 8).

## 4 DISCUSSION

The paracrine interplay between incretins and amines released from different enteroendocrine cell types acting upon surrounding epithelia and other resident cells within the submucosa, are not well understood. Recent morphological and histochemical studies of 5‐HT‐containing EC cells highlighted their regional differences in cell densities, distributions, the closed or open cell phenotype and their juxta‐position to enteric neurons in the mouse GI tract.^3^, ^41^ Additionally, our understanding of EC cells' chemosensory capacities and their ability to modulate signaling that alters mucosal functions and motility, remains patchy. Lund et al.^15^ showed that in the mouse colonic EC cells express significant levels of GLP‐1R and GIPRs, and when stimulated both peptide mechanisms facilitated the release of endogenous 5‐HT. While GLP‐1 effects were consistent in the small and large bowel, GIP's effect was restricted to the colon where, notably, similar 5‐HT amplification also occurs in response to a range of microbial metabolites.^27^ Given the potential for differential modulation of endogenous 5‐HT signaling, we set out to establish the sidedness and area‐specific differences in the amine's mucosal neuro‐epithelial responses and its mediation of GLP‐1 and GIP mucosal and motility activities in selected GI regions.

Basolateral 5‐HT_4_ receptor agonism resulted in rapid, consistent increases in epithelial ion transport along the length of the mouse GI tract, with an additional neuronal 5‐HT_3_ secretory component that was significant but restricted to the ascending (proximal) colon. In distal colonic mucosa the 5‐HT_4_ signals were TTX‐insensitive, providing further evidence that this receptor is unlikely to be located on submucosal neurons innervating this mucosal region in the mouse (Figure 8).

Epithelial 5‐HT responses have been described previously e.g. 5‐HT_4_‐mediated bicarbonate secretion in mouse duodenum,^42^ which maybe a protective buffering mechanism against gastric acid. Additionally, epithelial 5‐HT_4_ agonism elevates epithelial Cl^−^ secretion across colonic^43^ and caecal mucosae.^44^ The caecum also displayed a 5‐HT_3_‐mediated component, as we observed in the adjacent region of ascending colon. In all these studies, 5‐HT agonism was observed after basolateral administration. In the rat ascending colon, however, apical 5‐HT_4_ agonism caused epithelial bicarbonate secretion^32^ albeit at 10–100 times the agonist concentration we used. Apical 5‐HT is also reported to stimulate anion secretion via a 5‐HT_4_ mechanism, but only in full thickness preparations of mouse (Swiss Webster) colon where both enteric ganglionic networks remained intact,^33^ indicating intraluminal delivery of 5‐HT ligands may have therapeutic potential alongside reduced systemic side effects. However, we recorded minimal electrogenic responses after apical 5‐HT administration, ≤10% the size of the basolateral response in preparations where the submucosal innervation remained intact, but the myenteric innervation and smooth muscle layers had been removed. Apart from the difference in mouse strain and the muscle‐stripped preparations that we use to minimize barriers and maximize access to basolateral targets, it is unclear why such differences in 5‐HT response sidedness exist between our studies. In terms of the electrogenic output, our observations indicate that luminal 5‐HT exerts a minor effect upon ion transport. It is however possible that other electrically silent ionic transport processes or mucus secretion^33^ may result from luminally presented 5‐HT.

It is also noteworthy that the efficacy of exogenous 5‐HT responses was greatest in the mouse ascending colon compared with other regions, and this response pattern correlated with the mucosal 5‐HT levels measured using a boron‐doped diamond electrode in the same intestinal regions. The reductions in *I*_sc_ observed following basolateral administration of 5‐HT_3_ and 5‐HT_4_ antagonists reveals significant levels of endogenous 5‐HT_3_‐ and 5‐HT_4_‐mediated secretory tone; potentially a consequence of the high levels of basal 5‐HT we measured, particularly in the duodenum. There was no involvement of 5‐HT_2_ receptor‐mechanisms in colonic mucosa. 5‐HT_4_ secretory tone has been reported in rat colon mucosa^45^ and as we noted, this tonic activity was insensitive to TTX. In contrast, the 5‐HT_3_ tone revealed by tropisetron was TTX‐sensitive and is thus likely to be neural (excitatory) in origin in the mouse proximal colon. Blockade of 5‐HT reuptake with fluoxetine raised *I*_sc_ levels and this rapid effect was abolished by pre‐treatment with 5‐HT_3_ and 5‐HT_4_ antagonists, further supporting significant tonic 5‐HT release and coincident 5‐HT_3_ and 5‐HT_4_ mucosal secretory activities that are GI region‐specific.

A hormonal candidate for endogenous stimulation of ECs is GLP‐1.^15^ In the present study, TPH1 was co‐expressed in EC cells with GLP‐1R. Increasing concentrations of the GLP‐1 agonist, exendin‐4, stimulated 5‐HT release in mouse small and large intestinal regions. Previous studies have shown that GLP‐1 agonism stimulates the sensory neurotransmitter and secretagogue, calcitonin gene‐related peptide release from submucous nerves,^40^ which may account for the residual 5‐HT antagonist‐insensitive component of exendin‐4 responses in ascending colon mucosa (Figure 4). GLP‐1 is co‐expressed with PYY in L cells and area‐specific tonic mucosal activities have been characterized. Significant endogenous GLP‐1 tone^40^, ^46^ and PYY Y_1_ (and Y_2_) tonic activities occur in mucosae from normal mouse and human colon.^36^, ^39^, ^47^ In the current study 5‐HT mediated ~50% of the GLP‐1 responses and co‐localisation between TPH1 and GLP‐1R was evident in small and large intestine. The more complex GIP colonic responses exhibited initial secretory followed by anti‐secretory components, the former being 5‐HT‐mediated while the latter was PYY‐mediated (Figure 8). GIP‐stimulated PYY release was first identified in vascularly perfused but isolated rat colon.^48^ In the distal colon mucosa, where EC and L cells comprise the overwhelming majority of EECs, we observed a combination of 5‐HT_4_ and PYY (Y1 and Y2) antagonists abolished mucosal GIP responses. Thus, we annotate EC and L cells with basolateral GIP‐R, activation of which can stimulate endogenous 5‐HT, GLP‐1 and PYY epithelial responses in colonic mucosa (Figure 8). In the small intestine, GIP is contained predominantly in K cells, that are particularly abundant in the duodenum.^49^ While we observed significant co‐localisation of GIPR in colonic EC cells, this was not replicated in the duodenum, where GIP had no effect upon 5‐HT release. However, in functional *I*_sc_ studies, we did observe a transient 5‐HT component to basolateral hGIP administration in mucosae from both intestinal regions indicating that, although infrequent, sufficient GIPRs appear to be activatable by exogenous hGIP and can cause electrogenic epithelial responses. Unfortunately, in this study, we were unable to quantify the frequencies of peptide receptor/TPH1 co‐localisation in the small and large intestine and further imaging studies will be necessary to establish the relative abundance of GIPR (and GLP‐1R) on EC cells. Elevated plasma GIP levels following gastric emptying and stimulation of small intestinal K cells may be partly responsible for the PYY‐mediated aspects of ileal, and also colonic brakes,^50^ however, some prosecretory and potentially promotile 5‐HT activities are also a possible consequence of colonic GIPR stimulation.

The inter‐connections between mucosal ion transport and motility remain unclear. The mucosal lining provides multiple modulators of extrinsic^51^ and intrinsic colonic neural activities^20^; however, 5‐HT's mediation of these chemosensory effects remains controversial in terms of peristalsis.^20^, ^21^, ^22^ In addition, the cocktail of mediators most likely varies between GI regions (as we describe for endogenous 5‐HT secretory tone). This was borne out by a recent optogenetic study showing that distal, but not proximal colonic epithelial 5‐HT (and ATP) can initiate local and distant neuromuscular motility patterns.^52^ Notwithstanding the mechanistic limitations, we initially established 5‐HT_4_‐mediated natural fecal pellet propulsion. 5‐HT's promotile effects are well documented and known to activate circular muscle contraction, migrating myoelectric complexes and activation of descending interneurons.^4^, ^5^ 5‐HT_4_ agonists such as tegaserod are proven prokinetics developed to treat constipation, although their cardiovascular side‐effects terminated therapeutic use.^23^ GI‐restricted agonists would be advantageous^5^ but our mucosal studies showed clear basolateral‐predominant 5‐HT signaling, thus indicating the requirement for drug absorption for efficacy at epithelial or sub‐epithelial targets. Our isolated colon motility study did not discriminate luminal from circulating or bath applied drugs, but we assume that within the 20–40 min equilibration period drugs can access both lumen and lamina propria compartments. 5‐HT_4_ antagonists slowed natural fecal pellet excretion significantly in isolated WT and PYY^−/−^ colon, as well as in WT mouse whole GI transit assays. As PYY mediates ileal and colonic brakes^36^ PYY^−/−^ mice were used to more clearly discern any interplay between GLP‐1R and GIPR‐mediated anti‐motility effects. Exendin‐4 and GIP slowed fecal pellet transit and both responses were reversed by the GLP‐1 antagonist exendin (9–39) indicating that GIP's anti‐motile effect depends on GLP‐1 acting on GLP‐1R in the mouse colon, in a similar manner to the mucosal mechanisms (Figure 8). GLP‐1 agonism significantly slowed whole GI transit too (whereas GIP was ineffective) as did the combination of 5‐HT_3_ and 5‐HT_4_ antagonists, tropisetron and RS39604.

In conclusion, we have uncovered functional interactions between two major incretins and enteroendocrine 5‐HT‐mediated secretory and promotile effects. In distal colonic mucosa, GLP‐1 released from L cells and circulating GIP can each activate EC cells to enhance 5‐HT‐mediated release and local activities as proposed by Lund et al.^15^ In addition, we highlight the GI regional differences in 5‐HT levels, tonic 5‐HT activity, and the stimulated 5‐HT mechanisms that are predominantly basolateral in origin in healthy intestinal and colonic mucosae.

## AUTHOR CONTRIBUTIONS

Helen M. Cox designed the research with input from Thue W. Schwartz and Bhavik A. Patel. Iain R. Tough and Mari L. Lund performed the experiments and all authors contributed to the writing of the paper. All authors reviewed the final manuscript.

## CONFLICT OF INTEREST STATEMENT

The authors declare that they have no competing interests.

## Supporting information


Figure S1

Appendix S1
