# Supplementary figures and images for: Paracrine relationship between incretin hormones and endogenous 5‐hydroxytryptamine in the small and large intestine
Source: Neurogastroenterol Motil. 2023 Apr 3;35(8):e14589. doi: 10.1111/nmo.14589 (PMC10909488; doi:10.1111/nmo.14589)

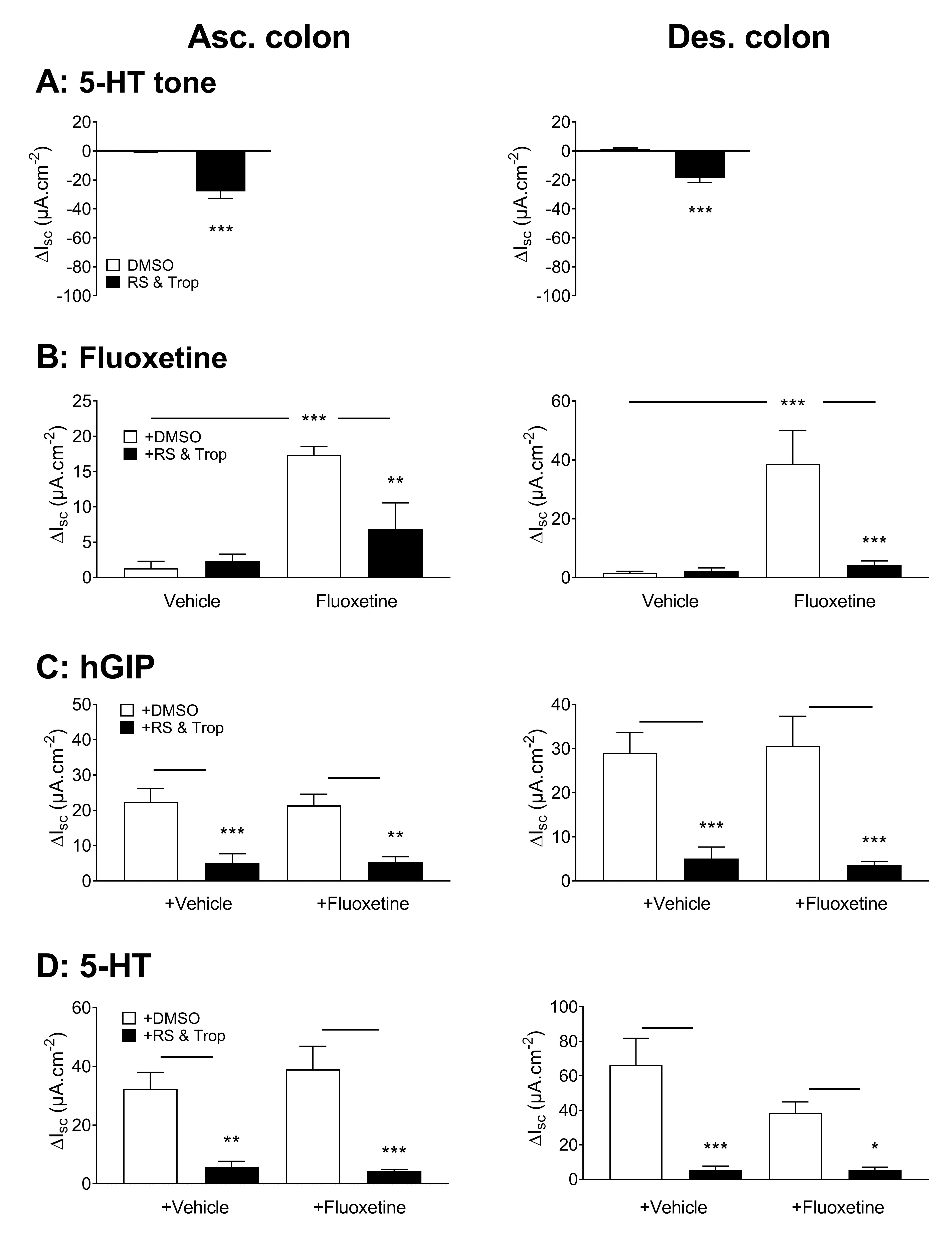

Supplement: Supplementary file 1 — Figure S1 [file NMO-35-e14589-s002.jpg]
