# Supplementary material for: Paracrine relationship between incretin hormones and endogenous 5‐hydroxytryptamine in the small and large intestine
Source: Neurogastroenterol Motil. 2023 Apr 3;35(8):e14589. doi: 10.1111/nmo.14589 (PMC10909488; doi:10.1111/nmo.14589)
Supplement: Supplementary file 2 — Appendix S1 [file NMO-35-e14589-s001.docx]

**Supporting Information:**

*SSRI with fluoxetine in ascending and descending colon mucosa*

Since hGIP responses appeared to involve both 5-HT and PYY activities (the former providing a secretory component, while PYY invoked anti-secretory effects) we tested the potential of the SSRI, fluoxetine to enhance endogenous 5-HT responses in PYY-/- ascending and descending colon mucosae. In both these colonic regions fluoxetine raised I_sc_ levels significantly revealing tonic endogenous 5-HT activity that was abolished by pretreatment with 5-HT_3_ and 5-HT_4_ antagonists (Figure S1A & B). GIP induced 5-HT responses were unaffected by the presence of fluoxetine (Figure S1C) while subsequent 5-HT responses were abolished by 5-HT_3_ and 5-HT_4_ antagonism (Figure S1D).
